# Consanguinity and rare mutations outside of MCCC genes underlie nonspecific phenotypes of MCCD

**DOI:** 10.1038/gim.2014.157

**Published:** 2014-11-06

**Authors:** Peter J. Shepard, Bruce A. Barshop, Matthias R. Baumgartner, John-Bjarne Hansen, Kristen Jepsen, Erin N. Smith, Kelly A. Frazer

**Affiliations:** 1 Department of Pediatrics, Rady Children’s Hospital, University of California, San Diego, La Jolla, California, USA; 2 Clinical and Translational Research Institute, University of California, San Diego, La Jolla, California, USA; 3 Division of Metabolic Diseases and Children’s Research Center, University Children’s Hospital Zurich, Zurich, Switzerland; 4 Zurich Center for Integrative Human Physiology, University of Zurich, Zurich, Switzerland; 5 Department of Clinical Medicine, University of Tromsø, Tromsø, Norway; 6 Institute for Genomic Medicine, University of California, San Diego, La Jolla, California, USA

**Keywords:** consanguinity, 3-methylcrotonyl-CoA carboxylase deficiency, newborn screening, runs of homozygosity

## Abstract

**Purpose:**

3-Methylcrotonyl-CoA carboxylase deficiency (MCCD) is an autosomal recessive disorder of leucine catabolism that has a highly variable clinical phenotype, ranging from acute metabolic acidosis to nonspecific symptoms such as developmental delay, failure to thrive, hemiparesis, muscular hypotonia, and multiple sclerosis. Implementation of newborn screening for MCCD has resulted in broadening the range of phenotypic expression to include asymptomatic adults. The purpose of this study was to identify factors underlying the varying phenotypes of MCCD.

**Methods:**

We performed exome sequencing on DNA from 33 cases and 108 healthy controls. We examined these data for associations between either MCC mutational status, genetic ancestry, or consanguinity and the absence or presence/specificity of clinical symptoms in MCCD cases.

**Results:**

We determined that individuals with nonspecific clinical phenotypes are highly inbred compared with cases that are asymptomatic and healthy controls. For 5 of these 10 individuals, we discovered a homozygous damaging mutation in a disease gene that is likely to underlie their nonspecific clinical phenotypes previously attributed to MCCD.

**Conclusion:**

Our study shows that nonspecific phenotypes attributed to MCCD are associated with consanguinity and are likely not due to mutations in the MCC enzyme but result from rare homozygous mutations in other disease genes.

## INTRODUCTION

3-Methylcrotonyl-CoA carboxylase (MCC) deficiency (MCCD) is a recessive disorder caused by homozygous or compound heterozygous mutations in either *MCCC1* or *MCCC2*. Each encodes a subunit of MCC, a biotin-dependent enzyme in the catabolic pathway of leucine.^1^ Clinical expression of MCCD has highly heterogeneous clinical symptoms; some individuals present with developmental delay, intellectual disability, neonatal seizures, ketoacidosis, cerebral edema, coma, or early death, whereas others are asymptomatic into adulthood.^2–5^ Our previous studies have shown that the causative mutations are located throughout the entire coding and some intronic sequences of *MCCC1* and *MCCC2*, resulting in a spectrum of genetic lesions including nonsynonymous, nonsense, splicing, and frameshift mutations. Approximately half of the individuals with MCCD are homozygous for the causative mutation, whereas the other half has compound heterozygous genotypes. Interestingly, neither the type of genetic lesion nor the status of homozygous or compound heterozygous mutation appears to be associated with severity of clinical symptoms.^6^

Although children with MCCD were previously identified during evaluation of developmental disability, metabolic disturbances, or Reye syndrome,^7^ newborn screening by tandem mass spectrometry (targeting 3-hydroxyisovalerylcarnitine as C5OH-carnitine)^8^ in the United States and a number of other countries around the world has resulted in greatly increased ascertainment of MCCD, with an estimated prevalence between 1:41,700 and 1:84,700.^9,10^ Before its inclusion in newborn screening programs, there were only ~30 reported cases of MCCD.^11^ Surprisingly, MCCD was found to be the third most common organic acid disorder detected in the newborn screening program in California^10^; however, results of a comparative analysis of case reports with newborn screening data suggest that less than 10% of affected individuals ever develop minor symptoms and less than 1 to 2% have a more severe outcome.^11^ When symptoms in an infant are ascertained through newborn screening, it is currently not possible to estimate clinical risk, and thus he/she is frequently administered lifelong treatment regimens including low-protein dietary modification and carnitine supplements. Because symptomatology is rarer than previously thought, prior to newborn screening programs, it is important to determine why some individuals show variable clinical symptoms and others do not. The risk–benefit balance of newborn screening is complicated because there are potential risks involved with too little intervention (not screening or not treating cases that will become symptomatic) or too much intervention (possibly resulting in unnecessary treatment, insurance implications, stigmatization, or promotion of anxiety in asymptomatic cases).

Recent reports have proposed that the attribution of nonspecific symptoms such as intellectual disability, attention deficit disorders, and fatigue to MCCD is questionable^10,11^ and may be caused by genetic variation outside of *MCCC1* and *MCCC2*.^12^ A number of MCCD patients have been previously reported to have consanguineous parents,^12^ which is associated with a wide variety of health and physical defects attributable to increased numbers of deleterious recessive alleles in the homozygous state. However, using self-reported family histories there was no significant association between parental consanguinity and the presence or absence of symptoms.^12^ Because of the importance of understanding the genetic components that underlie the diverse clinical manifestations of MCCD, in the present study we have used whole-exome sequencing data to directly investigate the role of genetic ancestry and consanguinity.

## MATERIALS AND METHODS

### Cohort selection

All samples were derived from remainders of specimens sent for clinical testing. Although many infants with MCCD that is ascertained through newborn screening appear to be healthy, it is not clear that they will remain asymptomatic. Accordingly, we analyzed 18 asymptomatic mothers who were discovered to have MCCD only by detection of abnormal C5OH-carnitine in the newborn screening sample from their healthy babies in whom the acylcarnitine elevation resolved. With the exception of subject A16, who had been previously reported,^6^ all other samples from asymptomatic individuals in this study were leftover aliquots of de-identified samples from adult females referred to our laboratory for diagnostic enzyme assay with clinical history as indicated. Lymphocytes derived from these 18 individuals were assayed and determined to have a very profound deficiency of MCC. Samples were also studied from 15 patients who were previously reported to be symptomatic,^2–4,6,13–22^ some of whom exhibited specific symptoms that are direct biochemical correlates of defective leucine catabolism,^23^ whereas others were reported to have a wide range of nonspecific symptoms (as outlined in Supplementary Table S1 online). Specific symptoms included ketoacidosis, hypoglycemia, hyperammonemia, coma, and plasma carnitine depletion with gross elevation of hydroxyisovaleryl-carnitine. Nonspecific symptoms, which are not directly related to leucine metabolism, included developmental delay, intellectual disability, seizures, hemiparesis, muscular hypotonia, and multiple sclerosis (Supplementary Table S1 online). We separated the MCCD cases into three groups, with group 1 containing 18 asymptomatic individuals, group 2 containing 5 individuals manifesting specific symptoms, and group 3 containing 10 individuals manifesting only nonspecific symptoms. One hundred eight healthy individuals of Norwegian ancestry from the Tromsø cohort^24^ were used as controls. The University of California, San Diego, Institutional Review Board (Human Research Protection Program) exempted the project under 45 CFR 46 subpart A §46.101b, category 4.

### Whole-exome sequencing

DNA (2.5 µg) was fragmented to ~175 bp using sonification (Covaris, Woburn, MA), fragment ends were repaired, and adaptors including sample index barcodes were ligated. The resulting DNA libraries were amplified by six cycles of PCR, 500 ng enriched using SureSelect Human All v4 kit (Agilent, Santa Clara, CA) to capture 51 Mb of target sequence,^25^ and sequenced (paired-end 100-bp reads) using a Illumina HiSeq 2000 (Illumina, San Diego, CA) (~100 million reads per sample; ~30× coverage). Reads were aligned to the human reference genome (hg19) using Burrows-Wheeler Aligner (BWA)^26^; read pairs identified as duplicates by Picard Mark Duplicates (http://sourceforge.net/projects/picard/) were removed. Using Genome Analysis Toolkit (GATK),^27^ reads were realigned around insertion/deletion sites and base quality scores were recalibrated. Genotypes were called in all 140 samples simultaneously using GATK and filtered using the variant quality score recalibration protocol.

Exome sequence data are available through dbGaP accession phs000776. http://www.ncbi.nlm.nih.gov/projects/gap/cgi-bin/study.cgi?study_id=phs000776.v1.p1.

### Identity by descent estimation

Identity by descent was estimated using PLINK (v1.07)^28^ using autosomal single-nucleotide polymorphisms (SNPs) with genotyping call rates >0.99, minor allele frequency >5%, and a per-sample call rate >80%. Pruning was performed to retain SNPs in linkage equilibrium at *r*^2^ value < 0.2.

### Ancestry estimation

Ancestry was estimated as previously described^29^ using the reference population of 1,445 unrelated participants of the 1000 Genomes Project (1KG) comprising five superpopulation groups (African, Asian, South Asian, European, and American admixed). Genotypes of these individuals on the Illumina Omni 2.5 array (Illumina, San Diego, CA) were downloaded (29 August 2012), and variants were linked to dbSNP 135 identifiers using GATK (there were 41,572 SNPs shared between the Omni 2.5 array and the exome sequencing data set). To identify SNPs informative for ancestry, informativeness (*I*_n_) was calculated across the five superpopulation groups, and markers were chosen in order of *I*_n_ that were in low linkage equilibrium (*r*^2^ < 0.2) with previously chosen markers within 1 Mb. SNPs with positive informativeness (*n* = 29,973) were used to cluster the genotypes of each MCCD case with the 1KG participants using multidimensional scaling in PLINK v1.07. To identify the most similar superpopulation for each MCCD case, a linear discriminant model was created based on the top 20 multidimensional scaling components using linear discriminant analysis (lda command in MASS package^30^ in R) with the 1KG individuals as a training set.

### Identification and analysis of runs of homozygosity

We identified long stretches of homozygous DNA (runs of homozygosity (ROHs)) using PLINK v1.07 (--homozyg) with a sliding window of 100 SNP length and a minor allele frequency criterion of 0.05 across MCCD and Tromsø individuals. The window threshold to call ROHs was 250 kb, and default values for the number of heterozygous SNPs (=1) and number of missing SNPs (=5) allowed in the ROH were used. To ensure that locally low SNP density did not spuriously increase the length of an ROH, PLINK v1.07 default values were used with a minimum SNP density of 50 kb and a maximum gap between two consecutive SNPs of 1,000 kb. To determine if the numbers of individuals with large ROHs in the three MCCD groups were statistically different, for each individual all regions larger than 1,600 kb were summed, and a Wilcoxon rank sum test was used to perform pairwise comparisons between groups 1, 2, and 3 and the Norwegian controls.

### Identification and annotation of high-impact variants in OMIM genes

Variant Call Format (VCF) files were annotated using SnpEff software^31^ and only high-impact (missense, frameshift, or splice site mutations) or moderate-impact (nonsynonomous mutations) variants were kept. Variants were further required to satisfy four criteria: (i) in a homozygous region ≥1.6 Mb in length; (ii) in a gene with an OMIM entry (http://omim.org/); (iii) either not present or at a frequency less than 1% in dbSNP (v137); and (iv) occurring in the exon or splice site of the OMIM gene according to the UCSC Genome Browser mRNA track. For Table 1, gene variants were annotated relative to transcripts NM_020166.3 for *MCCC1* and NM_022132.4 for *MCCC2* using Variant Effect Predictor (VEP).^32^ Gene variants are reported in accordance with the Human Genome Variation Society (http://www.hgvs.org/).

## RESULTS

### Whole-exome sequencing reveals no association between the type of genetic lesion or the status of homozygous/compound heterozygous mutations and the severity of clinical symptoms

To examine the genetic components underlying the varying clinical phenotypes associated with MCCD, we performed whole-exome sequencing identifying 274,496 and 307,935 variants (single-nucleotide variants and insertion/deletions combined) in the 33 cases and 108 controls, respectively. We first analyzed the mutational spectrum of the *MCCC1* and *MCCC2* genes in the 33 cases and observed that 15 individuals had mutations in *MCCC1* and 18 in *MCCC2* (Table 1). Fifteen cases were homozygous (6 for *MCCC1* and 9 for *MCCC2*), and 18 were compound heterozygous (9 for *MCCC1* and 9 for *MCCC2*). We observed 35 independent mutations, of which 8 mutations were observed more than once, resulting in the following patient pairs sharing a mutation: A5 and S20, A7 and A11; A9 and A19, A18 and A21; S8 and S25; S9 and S10; S4 and A5; S15, S22, and A20 (Table 1). Of the 35 mutations, 14 are reported in dbSNP (v137) with frequencies <0.0015, which is consistent with *MCCC1* and *MCCC2* null mutations being very rare in the population. For one case (A4) we identified a homozygous mutation 4 bp after an intron/exon boundary in *MCCC1*, suggesting this mutation may affect splicing. For seven cases we identified only one mutant allele (A13, A14, A18, A23, S9, S10, and S23) and inferred that they were compound heterozygotes because all individuals were shown to have enzyme deficiency, which is not the case when only a single allele is mutated.^33^ Consistent with previous reports,^6,12^ there was no apparent association between the type of genetic lesion or the status of homozygous or compound heterozygous mutations and the absence or presence/severity of clinical symptoms.

### MCCD cases that share the same mutation are likely to have inherited the variant from a common ancestor

For the MCCD cases that shared a common mutation, we checked for relatedness to determine if the mutations were inherited from a common ancestor. We estimated the proportion of the genome shared identically by descent using PLINK (PI_HAT value). We did not observe individuals related at the level of second-degree relatives or closer because all pairs of individuals were reported as having PI_HAT <0.25. Half of the pairs of individuals who shared a mutation had cryptic relatedness: A18 and A21 (*MCCC1* c.1526delC mutation) had a PI_HAT = 0.13, indicating third-degree relatives; S4 and A5 (*MCCC1* c.1155A>C mutation) had a PI_HAT of 0.09, indicating fourth-degree relatives; A5 and S20 (*MCCC1* c.974T>G mutation) have a PI_HAT of 0.09, indicating fourth-degree relatives; A9 and A19 (*MCCC1* c.558delA mutation) had a PI_HAT of 0.07, indicating fourth-degree relatives; and S22, S15, and A20 (*MCCC2* c.517_518insT mutation) had pairwise PI_HAT of 0.09 (Supplementary Table S2 online). For the three pairs of MCCD cases that shared an *MCCC2* mutation but did not have cryptic relatedness, we examined the genotypes of 36 SNPs (minor allele frequency >5%) in the 265-kb interval encompassing the *MCCC2* gene for identity by state (Supplementary Table S3 online). If the mutation had been inherited from the same distant relative, one would expect them to share not only the variant but also the surrounding chromosomal region. Cases S25 and S8 (*MCCC2* c.295G>C mutation) had identical genotypes at all 36 SNPs, indicating identity by descent. Cases A7 and A11 (*MCCC2* c.1065A>T mutation) had identical genotypes at 35 of the 36 SNPs, indicating identity by descent. S9 and S10 (both compound heterozygotes for *MCCC2* c.1015G>A mutation) had dissimilar genotypes in the proximal part of the interval but had three SNPs (spanning 5.5 kb) immediately proximal to the mutation and 21 SNPs (spanning ~800 Kb) distal to the mutation in which one allele was shared in common, suggesting the mutation arose only once in a distant ancestor. Our data demonstrate that MCCD cases that share the same mutation are most likely to have inherited the variant from a common ancestor.

### Genetic ancestry does not seem to be an important determinant of the diverse clinical manifestations

**Figure 1:**
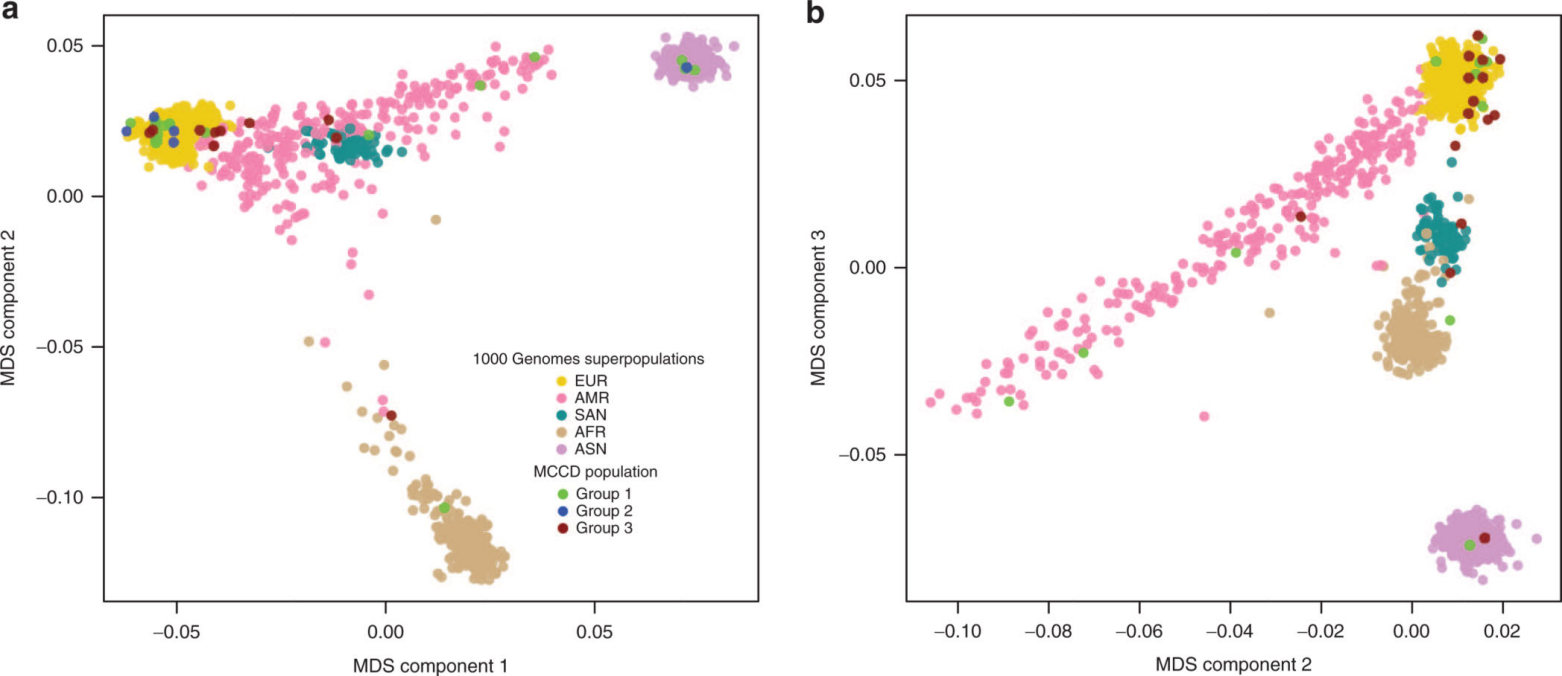
Genetic clustering of participants in the 1000 Genomes Project and MCCD individuals One thousand four hundred forty-five unrelated individuals from the 1000 Genomes Project and 33 individuals from the MCCD population were clustered on genotype profiles using multidimensional scaling (MDS) and plotted according to their final scores on the (**a**) first two dimensions and (**b**) the second and third dimensions. European (EUR), African (AFR), and Asian (ASN) 1000 Genomes Project superpopulation groups are clearly differentiated in the first two dimensions, whereas South Asian (SAN) and American admixed (AMR) groups are overlapping, reflecting their historical European and Asian ancestry. The individuals in the SAN group cluster together. The AMR group is broadly distributed, indicating that some individuals are genetically more similar to the EUR group than others to either the ASN or AFR groups. In the second and third dimensions, SAN and AMR are clearly and distinctly identifiable. MCCD, 3-methylcrotonyl-CoA carboxylase deficiency.

To determine whether ethnicity plays a role in the manifestation of different clinical phenotypes, we estimated the genetic ancestry of each MCCD case using the 1KG superpopulations (see Materials and Methods). We visualized ancestry estimation for all cases by plotting the first versus second multidimensional scaling components (Figure 1a) and the second versus third components (Figure 1b). We had self-reported ancestry for 11 individuals and observed high agreement between reported ancestry and clustering with expected 1KG superpopulations (Supplementary Table S4 online). Additionally, the seven pairs of individuals who inherited the same mutation from a common ancestor had similar estimated genetic ancestry. We observed that there are approximately equal numbers of asymptomatic (group 1) and symptomatic MCCD individuals (groups 2 and 3) assigned to each of the five 1KG superpopulations. Thus, genetic ancestry does not seem to be an important determinant of the diverse clinical manifestations observed among individuals with MCCD.

### Association of consanguinity with MCCD clinical phenotypes

**Figure 2:**
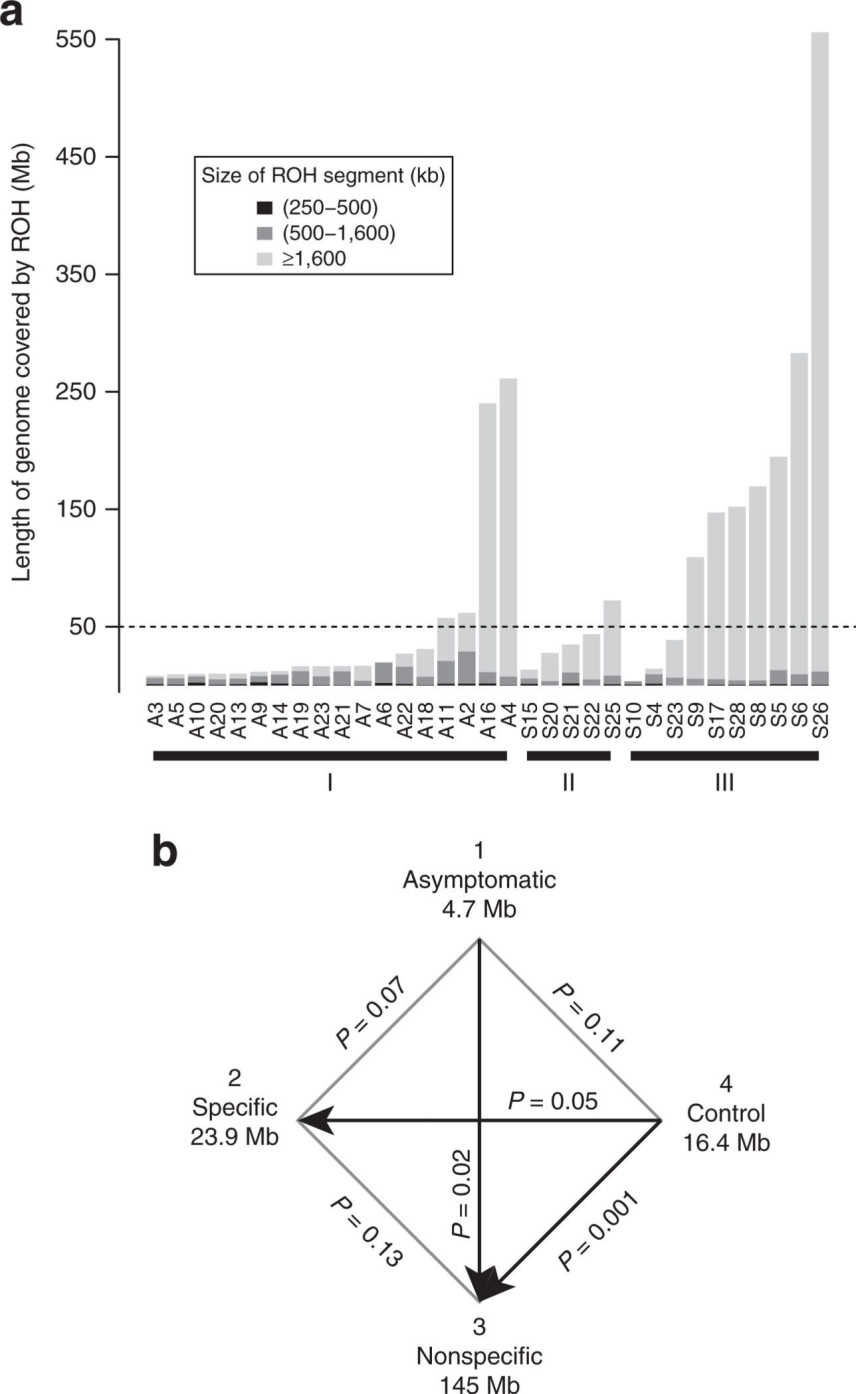
Comparison of amount and type of ROH for MCCD groups 1, 2, and 3 (**a**) For each individual, the summed totals of ROHs for the three classes (250–500 kb, 500–1,600 kb, >1,600 kb) are shown for MCCD individuals organized by symptom group. The dashed line indicates the expected amount of homozygosity that would result in the union of second cousins. (**b**) Pairwise *P* values from the comparison of total ROHs >1,600 kb between each MCCD group and Tromsø control group using a Wilcoxon rank sum test. Below each group is the median total length of ROH segments >1,600 kb for the individuals in each group. MCCD, 3-methylcrotonyl-CoA carboxylase deficiency; ROH, run of homozygosity.

To examine whether consanguinity is associated with MCCD clinical phenotypes, for each case we assessed the amount and characterized the length of autosomal DNA segments that were homozygous (runs of homozygosity, ROHs). ROHs are common in human genomes and represent segments in which identical haplotypes are inherited from each parent. Pemberton et al.^34^ recently classified ROHs by length: short ROHs, which reflect ancient haplotype blocks; intermediate ROHs, which reflect background relatedness attributable to a population bottleneck; and large ROHs, attributable to recent parental relatedness. For each case, ROHs were categorized into three size classes: short regions (250–500 kb), medium regions (500–1,600 kb), and large regions >1,600 kb (Figure 2a). Four (22%) group 1 cases, one (20%) group 2 case, and seven (70%) group 3 cases have ROHs at or greater than the level anticipated for individuals who are the product of second cousin unions. We performed all pairwise comparisons among the three MCCD groups to test whether the groups differed by their total length of large ROHs (>1,600 kb) using a Wilcoxon rank sum test (Figure 2b). We show that MCCD group 3 has significantly higher levels of homozygosity than MCCD group 1 (*P* = 0.02). Although the data suggest that group 3 has higher median levels of homozygosity than group 2 and that group 1 had lower median levels than group 2, the test failed to achieve significance (*P* = 0.13 and *P* = 0.07, respectively), possibly because of a small sample sizes. Recent studies show that ROHs are more common and longer than expected in unrelated individuals from outbred populations.^35,36^ For this reason we analyzed the 108 controls (Supplementary Figure S1 online), which revealed that only 5 (5%) of these individuals had ROHs at the level consistent with being the product of a second cousin union. Comparisons of each MCCD group with the healthy control group using the Wilcoxon rank sum test demonstrate that group 2 and group 3 MCCD cases had higher median homozygosity than the controls, but only group 3 showed a significant difference (*P* = 0.001). Our results show that group 3 cases are significantly more likely to be inbred compared with cases that are asymptomatic and healthy controls.

### Rare mutations outside of MCCC genes underlie nonspecific phenotypes of MCCD

We hypothesized that group 3 cases harbor homozygous recessive mutations in disease genes other than *MCCC1* or *MCCC2* that are responsible for their nonspecific clinical phenotypes. We performed a genome-wide search for rare homozygous variants in large ROHs with predicted high or moderate impact on the function of a gene with an OMIM entry. For two patients, we observed novel mutations in disease genes and a striking number of the patients’ clinical symptoms matching the symptoms described in the OMIM entry. In S9, a 1-bp homozygous deletion results in a premature stop codon in immunoglobulin mu binding protein 2 (*IGHMBP2*) (Table 2). Homozygous mutations in *IGHMBP2* are known to cause spinal muscular atrophy type 1, which has clinical features matching those of S9 (Table 3). In S28, we identified a 1-bp homozygous deletion in aldehyde dehydrogenase 7 family, member A1 (*ALDH7A1*), which may better explain the reported neonatal seizures and medically resistant status epilepticus in this patient. For three patients, we identified rare variants (minor allele frequency <0.0015) that result in nonsynonomous substitutions or frame-shifts in OMIM disease genes. Although these mutations are in dbSNP, they occur less frequently than the *CFTR* p.Phe508del mutation (rs121909001; minor allele frequency ~0.015), which is the leading cause of cystic fibrosis. For S17, we observed a homozygous nonsynonomous variant in tetratricopeptide repeat domain 37 (*TTC37*), which may underlie the reported frequent watery diarrhea, fatigue, and failure to thrive in this individual. In S8, a homozygous nonsynonomous variant in aarF domain containing kinase 3 (*ADCK3*) may explain the reported progressive seizures and developmental delay. Finally, in S26 a homozygous variant resulting in a frameshift in solute carrier family 46 (folate transporter), member 1 (*SLC46A1*), may result in the reported dysmyelination and hemiparesis. Thus, for 50% of the MCCD cases in group 3, we observed a homozygous damaging novel mutation or rare variant in a disease gene that likely explains their clinical symptoms independently of their mutations in *MCCC1* or *MCCC2*.

## DISCUSSION

The recommendation from the American College of Medical Genetics and Genomics to include MCCD in the newborn screening core panel^37^ was based on availability and perceived necessity of treatment and good understanding of the natural history of the disease. Our study suggests that many of the nonspecific symptoms attributed to MCCD may not be caused by mutations in MCC, but rather are caused by mutations in other disease genes. Although having multiple rare recessive mutations is unusual, the probability of having any rare recessive disease is increased by consanguinity. Thus, it is not unusual that patients from consanguineous unions affected with MCCD also appear to be more likely to carry other rare mutations. Our results together with findings of other studies^9,33,38^ suggest that the clinical consequences of MCCD may be less pervasive than previously believed, and when present may be limited to specific effects of defective leucine catabolism. Further studies are needed to assess the value of providing treatment to asymptomatic individuals with MCCD who are identified through newborn screening.

Although our results help to explain the difference between MCCD patients with nonspecific and specific symptoms, they do not address why there are phenotypic differences between asymptomatic MCCD individuals and MCCD patients with specific symptoms. The biochemical context of MCC in leucine catabolism might arguably render its deficiency particularly likely to escape phenotypic expression. Substrate accumulation seems to be well tolerated to very high levels, and excretion of 3-methylcrotonylglycine, 3-hydroxyisovaleric acid, and 3-hydroxyisovalerylcarnitine is efficient (provided that there is no depletion of carnitine). Also, the impact of product limitation may be minimal because the product of MCC, 3-methylglutaconylCoA, can be generated by reversal of 3-methylglutaconyl-CoA hydratase, and the subsequent product, hydroxymethylglutaryl-CoA, may be alternatively formed from acetoacetate derived from fatty acid oxidation. Therefore, to observe a disease phenotype, it may be that mutations other than those in *MCCC1* and *MCCC2* are needed. Another possibility is that environmental factors (e.g., infection, catabolic stress) trigger specific symptoms in some MCCD patients. Further studies using cases specifically determined not to be inbred might help resolve the difference between asymptomatic MCCD cases and MCCD cases with specific symptoms.

Our study has implications for current undiagnosed disease studies that are attempting to correlate phenotypes to specific genotypes through large-scale sequencing. Because individuals from consanguineous unions carry rarer recessive mutations that could cause genetic diseases, these individuals may be more likely to be recruited to very rare disease studies. For the same reason, they would also be more likely to have phenotypes that could be due to multiple loci. Therefore, care must be taken in extrapolating that a mutation that explains a portion of the clinical phenotype is causal for the entire complex phenotype. Patients with symptoms beyond the usual spectrum of disease for a given disorder should be evaluated for other contributing alleles and putative phenotypic modifiers. Additionally, inbred individuals are often purposely used to identify disease genes due to the ease of mapping autosomal recessive disorders by identifying regions of the genome that are homozygous in all affected individuals.^39,40^ Our results suggest that both genotype–phenotype correlative sequencing projects and homozygosity mapping studies should take into account the level of inbreeding in individuals when attributing the effects of a shared mutation on a phenotypically heterogeneous group.

## Figures and Tables

**Table 1:** Mutation status of *MCCC1* and *MCCC2* genes and clinical data of 35 MCCD patients

Gene	Group	Sample	Sex	Mutation^a^	DNA change^b^^c^^d^	Protein change	Effect	Reference
*MCCC1*	1	A2	F	Homozygous	c.1315G>A	p.Val438Met	Nonsyn	This study
		A3	F	Compound Het.	c.1750C>T*; c.1894C>T	p.Gln584X; p.Pro632Ser	Stop; Nonsyn	This study
		A4	F	Homozygous	c.1731+4A>G	Intronic, splice region	UNK	This study
		A5	F	Compound Het.	c.1155A>C*; c.974T>G*	p.Arg385Ser; p.Met325Arg	Nonsyn; Nonsyn/splicing	This study
		A9	F	Compound Het.	c.841C>T; c.558delA	p.Arg281X; p.Gln186Hisfs*6	Stop/Nonsyn	This study
		A10	F	Compound Het.	c.1193_1194delTG; c.834A>G	p.Val397Glyfs*19; p.Gln129Arg	Nonsyn; Nonsyn	This study
		A18	F	Compound Het.	c.1526delG; UND	p.Cys509Serfs*14	Nonsyn	This study
		A19	F	Compound Het.	c.539G>T; c.558delA	p.Gly180Val; p.Gln186Hisfs*6	Nonsyn; Nonsyn	This study
		A21	F	Homozygous	c.1526delG	p.Cys509Serfs*14	Nonsyn	This study
		A23	F	Compound Het.	c.872C>T; UND	p.Ala291Val	Nonsyn	This study

	2	S20	M	Compound Het.	c.866C>T; c.974T>G*	p.Ala289Val; p.Met325Arg	Nonsyn; Nonsyn/splicing	Ref. 15

	3	S4	F	Homozygous	c.1155A>C*	p.Arg385Ser	Nonsyn	Refs. 15,16
		S5	F	Homozygous	c.1594G>C*	p.Asp532His	Nonsyn/splicing	Ref. 15
		S6	M	Homozygous	c.1310T>C*	p.Leu437Pro	Nonsyn	Refs. 4,15
		S26	F	Compound Het.	c.1114C>T; c.1882G>T	p.Gln372X; p.Glu628X	Stop; stop	Ref. 18

*MCCC2*	1	A6	F	Homozygous	c.592C>T	p.Gln198X	Stop	This study
		A7	F	Homozygous	c.1065A>T	p.Leu355Phe	Nonsyn	This study
		A11	F	Homozygous	c.1065A>T	p.Leu355Phe	Nonsyn	This study
		A13	F	Compound Het.	c.302C>T; UND	p.Ser101Phe	Nonsyn	This study
		A14	F	Compound Het.	c.214C>T*; UND	p.Arg72X	Nonsyn	This study
		A16	F	Homozygous	c.1367C>T	p.Ala456Val	Nonsyn	Ref. 6
		A20	F	Compound Het.	c.517_518insT; c.599T>A*	p.Ser173Phefs*25; p.Ile200Asn	Frameshift; Nonsyn	This study
		A22	F	Compound Het.	c.1488G>C; c.351_353delTGG	p.Gln496His; p.Gly118del	Nonsyn; in-frame deletion	This study

	2	S15	F	Compound Het.	c.517_518insT; c.994C>T	p.Ser173Phefs*25; p.Arg332X	Frameshift/stop; stop	Refs. 6,13,15
		S21	M	Compound Het.	c.464G>A*; c.929C>G*	p.Arg155Gln; p.Pro310Arg	Nonsyn; Nonsyn	Refs. 2,15
		S22	F	Homozygous	c.517_518insT	p.Ser173Phefs*25	Frameshift/stop	Ref. 15
		S25	F	Homozygous	c.295G>C*	p.Glu99Gln	Nonsyn	Ref. 15

	3	S8	M	Homozygous	c.295G>C*	p.Glu99Gln	Nonsyn	Refs. 3,15
		S9	M	Compound Het.	c.1015G>A*; UND	p.Val339Met	Nonsyn	Refs. 15,17
		S10	F	Compound Het.	c.1015G>A*; UND	p.Val339Met	Nonsyn	Ref. 15
		S17	F	Homozygous	c.1054G>A	p.Gly352Arg	Nonsyn/splicing	Refs. 6,20
		S23	M	Compound Het.	c.1309A>G*; UND	p.Ile437Val	Nonsyn	Refs. 14,15
		S28	M	Homozygous	c.116C>T	p.Ser39Phe	Nonsyn	Ref. 19

F, female; fs, frameshift; frameshift/stop, frameshift mutation that also results in a downstream stop codon; Het., heterozygous; M, male; MCCD, 3-methylcrotonyl-CoA carboxylase deficiency; Nonsyn, nonsynonymous; nonsyn/splicing, nonsynonymous mutation that also disrupts mRNA splicing; UND, undetermined; UNK, unknown.

a Mutation type: homozygous, both alleles are non-hg19 reference genome; compound Het., two heterozygous non-hg19 reference genome alleles.

b If only one allele is found in a compound heterozygote, then the second allele is shown as “UND” in the DNA change column.

c* Present in dbSNP at frequency less than 0.0015.

d Variants are reported with reference to transcripts NM_020166.3 for MCCC1 and NM_022132.4 for MCCC2.

**Table 2:** Mutations underlying the nonspecific clinical phenotypes of MCCD cases

Sample	Chromosome	Position	dbSNP ID	Global minor allele frequency^a^	Reference allele	Alternate allele	Effect	Gene symbol
S9	chr11	68703767	—	0	C	CA	Frameshift	*IGHMBP2*
S17	chr5	94857860	rs199854306	0.0014	T	C	Nonsynonomous	*TTC37*
S28	chr5	125880679	—	0	GC	G	Frameshift	*ALDH7A1*
S8	chr1	227171824	rs144147839	0.00022	A	G	Nonsynonomous	*ADCK3*
S26	chr17	26727721	rs5819844	0.00138	GA	G	Frameshift	*SLC46A1*

MCCD, 3-methylcrotonyl-CoA carboxylase deficiency.

a The global minor allele frequency is reported from dbSNP v.137 and is derived from the 1000 Genomes Project phase I data.

**Table 3:** OMIM entry disease descriptions matched with the symptoms of the corresponding MCCD case

Sample	Symptoms^a^	OMIM number	OMIM title	Matching terms
S9	Failure to thrive, hypotonia, diaphragmatic paresis and brain atrophy, progressive respiratory insufficiency, fatal at 6.5 months	604320	DISTAL SPINAL MUSCULAR ATROPHY, AUTOSOMAL RECESSIVE, 1	Muscular hypotonia, diaphragmatic paresis, respiratory failure
S17	Failure to thrive at age 7 months, frequent watery diarrhea, fatigue	222470	TRICHOHEPATOENTERIC SYNDROME 1; THES1	Failure to thrive, frequent watery diarrhea
S28	Neonatal seizure onset, unresponsive to standard anticonvulsants, status epilepticus	107323	EPILEPSY, PYRIDOXINE-DEPENDENT	Neonatal seizure onset, unresponsive to standard anticonvulsants, status epilepticus
S8	Developmental delay, muscular hypotonia, progressive seizures, died from circulatory failure after prolonged epileptic seizures	606980	AARF DOMAIN-CONTAINING KINASE 3; ADCK3	Developmental delay, progressive seizures
S26	Psychomotor developmental delay, encephalopathy at 5 years of age, hemiparesis at 13 years of age consistent with multiple sclerosis course, neutropenia	611672	SOLUTE CARRIER FAMILY 46 (FOLATE TRANSPORTER), MEMBER 1	Delayed motor development, delay in myelination, hemiplegic, leukopenia

MCCD, 3-methylcrotonyl-CoA carboxylase deficiency.

a Symptoms as reported by treating physician (references in Table 1).

## Footnotes

**SUPPLEMENTARY MATERIAL** Supplementary material is linked to the online version of the paper at http://www.nature.com/gim.
